# Editorial: Advances in the diagnosis and treatment in kidney transplantation

**DOI:** 10.3389/fmed.2022.967749

**Published:** 2022-08-03

**Authors:** Kathrin Eller, Georg A. Böhmig, Miriam C. Banas, Ondrej Viklicky

**Affiliations:** ^1^Division of Nephrology, Medical University of Graz, Graz, Austria; ^2^Division of Nephrology and Dialysis, Department of Medicine III, Medical University of Vienna, Vienna, Austria; ^3^Department of Nephrology, University Hospital Regensburg, Regensburg, Germany; ^4^Department of Nephrology, Transplant Center, Institute for Clinical and Experimental Medicine, Prague, Czechia

**Keywords:** transplantation, antibody-mediated rejection (ABMR), machine perfusion, kidney, cardiovascular disease, infection

## Introduction

**Figure 1:**
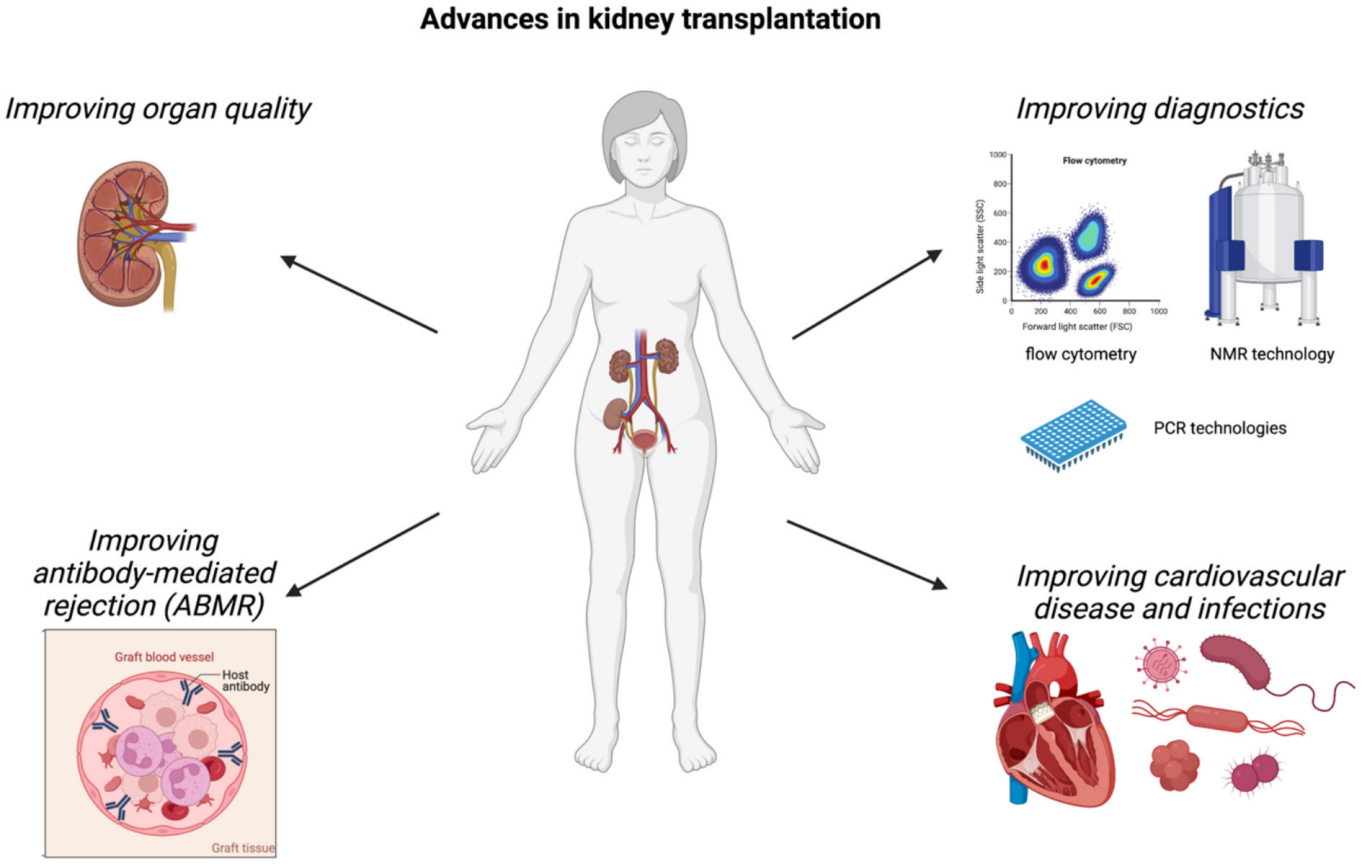
“*Advances in the Diagnosis and Treatment in Kidney Transplantation*.” This Research Topic provides new evidence in improvements in different fields of kidney transplantation. Articles published focus on the improvement of organ quality using machine perfusion, of diagnostic possibilities to detect rejection and graft function, of antibody-mediated rejection (ABMR) as well as cardiovascular and infection outcomes Created with BioRender.com.

Kidney transplantation significantly improves patient survival and quality of life in patients with chronic kidney disease (CKD) stage 5 and is thus considered as the current optimal therapy for this patient cohort. More than 21,000 kidney transplantations have been performed in the EU in 2019 (1). Even though advances have been made in treating kidney transplant recipients leading to an improved graft and patient survival despite of increased numbers of transplanted organs from extended criteria donors (2), further advancements are needed especially in the field of organ preservation/regeneration using machine perfusion, diagnostic evaluations, antibody-mediated rejections as well as improvement of cardiovascular disease and treatment of infections (Figure 1). We believe that these fields hold great potential to improve the quality of treatment of our patients after kidney transplantation leading to better patient and graft outcomes. In the Research Topic of Frontiers in Medicine “*Advances in the Diagnosis and Treatment in Kidney Transplantation*,” a great number of publications within these fields have been published and will be discussed in this Editorial.

## Strategies to overcome ischemia-reperfusion injury

Ischemia-reperfusion injury has been considered to be the inevitable consequence of every transplantation procedure. Both the advanced donor age which reflects the poor organ quality and longer ischemia time were shown to be associated with delayed graft function and inferior kidney grafts outcome. Therefore, several attempts have been done to overcome such an obstacle to improve graft quality and prolong time of preservation. Among those Moers et al. as early as in 2009 presented the first randomized clinical trial on machine perfusion and found that hypothermic perfusion was associated with a reduced risk of delayed graft function and improved graft survival at the 1st year after transplantation (3). Since that time many transplant centers have introduced this method which has become a routine for at least grafts at risk for delayed graft function. Zulpaite et al. reviewed therapeutic potential and challenges of *ex-vivo* kidney machine perfusion techniques in this Research Topic. Furthermore, two original clinical studies and one experimental study have been published. Zeng et al. performed a small study comparing traditional antegrade perfusion technique with retrograde approach when they cannulated either renal artery or renal vein for hypothermic LifePort Kidney Transporter perfusion. Authors found that both approaches are associated with similar delayed graft function rates and eGFR at 6 months. Clearly, the study was underpowered and future studies are necessary to show this approach may have some advantages over the classical approach. Next, Weissenbacher et al. evaluated hemodynamic and metabolic parameters in normothermic kidney preservation. Normothermic machine preservation has been studied by several groups and this method is about to enter the clinical medicine. Normothermic machine perfusion may eliminate the effect of cold ischemia and allows evaluation of function and metabolic status of the graft and thus may help to decide whether the organ is still suitable for transplantation (4). Authors used 12 discarded human kidneys which underwent normothermic machine perfusion for 24 h. In eight cases, urine recirculation was used and in four case urine was replaced by Ringer's lactate. Several biomarkers and parameters of machine perfusion were assessed. Arterial flow, pH, NGAL and L-FABP correlated with donor creatinine and eGFR. Perfusate TNF-α was higher in kidneys with lower arterial flow. The cytokines IL-1β and GM-CSF decreased during 6 h. Kidneys with more urine output had also lower perfusate KIM-1 levels. These parameters may be considered as additional viability markers.

Finally, Xiang et al. showed in the mouse model of kidney cold storage that in kidney proximal tubules the p53, a tumor suppressor and stress response gene, is associated with kidney injury and graft dysfunction. They demonstrated that pharmacological inhibition of p53 may reduce acute tubular injury, apoptosis and inflammation at 24 h after cold storage. These preclinical data support a role of p53 in the graft injury associated with ischemia-reperfusion injury and thus demonstrate the possible therapeutic potential of p53 inhibitors.

## Diagnostics in kidney transplantation

During the last years diagnostic procedures have significantly improved. Especially in the field of transplantation, important progress has been made in the areas of biomarker search and omics technologies. These are not limited to kidney biopsies, but also include non-invasive investigations using serum or urine.

The use of omics technologies has a relevant impact to the further development of diagnostic possibilities, not only in terms of finding a diagnosis but also in making a prognosis, such as transplant survival.

The molecular microscope developed by Halloran et al. makes an interesting contribution to the diagnosis of rejection and injury in transplanted organs (5). Using indication biopsies from the INTERCOMEX study (6) the actual study by Halloran et al. was designed to extend previous investigations and to classify scenarios associated with parenchymal injury. By using archetypal analysis (AA) of scores for gene sets and classifiers previously identified in various injury states, six injury groups were defined. Two classes of early AKI could be separated showing differences in function, parenchymal dedifferentiation, response to injury, inflammation and graft survival. The most important predictors of function (estimated glomerular filtration rate) and graft loss were injury-based molecular scores, not rejection scores.

Zero-time biopsies reflect the state of a donor organ before transplantation. In contrast to other organ transplants zero biopsies in kidneys are not routinely performed. Vonbrunn et al. investigated in 26 zero-time biopsies gene expression profiles for different types of subsequent renal transplant complications. They could reveal significant differences between living and deceased donor kidneys mainly due to differences in the cold ischemia time. Genes encoding for immunoglobulins were differentially expressed in biopsies from transplants which later developed rejection corresponding to higher number of CD20^+^ and CD138^+^ cells. Acute renal failure before transplantation also had an influence on gene expression. Although transplant biopsies are still the gold standard in routine diagnostic, non-invasive diagnostic from urine or plasma can help to identify patients at risk for renal transplant rejection. Analysis of donor derived cell-free DNA (dd-cfDNA) has been shown to have the potential to detect cases of rejection episodes, with patients with rejection having higher plasma dd-cfDNA levels than patients with stable grafts without rejection (7). In their study Jerič Kokelj et al. describe a novel method using droplet digital PCR analysis to quantify dd-cfDNA in kidney transplant patients. By using a novel pilot set of assays targeting single nucleotide polymorphisms they found that droplet digital PCR is suitable for analysis of kidney transplant patients' plasma but recommend prior genotyping of donor DNA and performing reliable preamplification of cfDNA. As long as the transplant patient has sufficient urine output it is possible to use it for diagnostic investigations. Oblak et al. could correlate the estimated protein excretion rate in the 1st year after kidney transplantation with ABMR, vascular TCMR and *de novo* DSA, although not surprisingly the test validity was higher in ABMR compared to TCMR (AUC 0.95 vs. 0.68). Previously Banas et al. developed a novel, non-invasive method to detect graft rejection *via* a characteristic constellation of urine metabolites by NMR spectroscopy (8). In the following prospective international PARASOL study Banas et al. collected 1,230 urine samples and matched them to the corresponding kidney transplant biopsies. The clinical characteristics of subjects recruited, indicate a patient cohort typical for European renal transplantation. A typical shift from T-cellular early rejections episodes to later antibody mediated allograft damage over time after renal transplantation further strengthens the usefulness of the cohort for the evaluation of novel biomarkers for allograft damage. Living-donor kidney transplant recipients undergoing desensitization for Human Leukocyte Antigen (HLA)-incompatibility have a high risk of developing antibody-mediated rejection (ABMR). The purpose of the study by Cucchiari et al. was to evaluate if residual B cell activity after desensitization could be estimated by the presence of circulating B cell-derived extracellular vesicles. In studies on patients before and after desensitization and controls they could demonstrate a significant drop in B cell-derived extracellular vesicles after desensitization and that this paralleled the reduction in CD19^+^ cells in lymph nodes, while in peripheral blood B cells, this change was almost undetectable.

To unravel the finding why kidney transplant recipients show impaired immune responses to SARS-CoV-2 infection and a reduced efficacy of SARS-CoV-2 vaccination compared to dialysis patients, Schuller et al. investigated peripheral blood B cell composition before and after kidney transplantation. They could detect persistent and profound compositional changes within the B cell compartment. Low Transitional B cells, 1 year after KT, may account for the low serological response to SARS-CoV-2 vaccination in KTRs compared to dialysis patients.

## Advances in antibody-mediated rejection

In the last two decades, antibody-mediated rejection (ABMR) has become a major research focus in transplant medicine, and, today, our knowledge about molecular mechanisms and the manifold phenotypic presentation of this rejection type has improved substantially. Nevertheless, there are large numbers of unanswered questions, some of them now addressed in the context of our present Research Topic. One is the burden of recipient sensitization—a dominant risk factor of ABMR occurrence. Individualized risk assessment in this context may be of decisive importance to adequately guide organ allocation and tailor the composition or intensity of desensitization protocols (9, 10). There is still a need for immunological variables that accurately predict allograft outcomes. In a retrospective cohort study including 108 donor-specific antibody (DSA)-positive deceased donor transplant recipients subjected to desensitization with anti-thymocyte globulin, plasmapheresis, intravenous immunoglobulin (IVIG) and/or rituximab, Osickova et al. found particularly high ABMR rates among recipients with a positive pre-transplant flow cytometric crossmatch (76 vs. 19% among crossmatch-negative recipients), demonstrating a 5-fold increased risk in multivariable analysis. These interesting results are of considerable clinical relevance, as they strongly support the systematic use of flow crossmatch testing in the context of DSA-positive deceased donor kidney transplantation. Moreover, Kälble et al. evaluated allograft outcomes in 38 DSA and/or crossmatch-positive recipients of a living donor transplant following an individualized algorithm of peri-transplant apheresis. Treatment, which included rituximab and/or thymoglobulin, was tailored according to careful immunological risk stratification, among others, based on single bead assay results and/or soluble CD30 (sCD30) monitoring. Patient and graft survival rates were found to be similar to those observed among standard-risk recipients, without differences regarding rejection rates. An interesting observation was that, following transplantation, 56% of the recipients had lost their DSA. ABMR rates in these patients were only 6%, but 60% in recipients with persistent and *de novo* DSA. In a retrospective study of 287 patients subjected to standard immunosuppression, Drasch et al. evaluated the impact of preformed DSA and sCD30 levels on renal allograft outcomes. In their study, graft survival was significantly lower in DSA-positive as compared to DSA-negative patients. While DSA-positive patients with increased levels of sCD30 had adverse 3-year graft survival, sCD30 levels were not associated with ABMR frequency, DSA persistence and long-term survival. Senn et al. studied a distinct risk constellation—husband-to-wife transplantations with mutual children—which may be complicated by the persistence of alloreactive T and B cells triggered by paternal HLA antigens. Analyzing 25 such transplants in comparison to women with prior pregnancies who received a kidney from other donor sources, they found numerically higher incidences of ABMR and inferior death-censored graft survival, despite the use of T cell-depleting induction therapy. Interestingly, in this cohort, DSA status, number of pregnancies, or the number of HLA-mismatches were not predictive for rejection or graft loss.

Treatment of ABMR has remained a major challenge, and evidence for efficacy of currently available anti-rejection treatments is considerably low, especially in chronic rejection. One promising concept of ABMR treatment may be the use of antibodies directed against interleukin-6 (IL-6) or its receptor (IL-6R). Noble et al. present a single-center study including 40 renal allograft recipients who all received the anti-IL-6R antibody tocilizumab for chronic active ABMR. Six patients lost their graft within 12 months, but many patients showed stable eGFR, and there was no change in microvascular inflammation scores and the extent of interstitial fibrosis and tubular atrophy. Transplant glomerulopathy scores, however, increased. Even though limited by its uncontrolled retrospective design, this study may suggest that tocilizumab is able to stabilize the decline in renal function and histological rejection lesions, at least in some of the treated patients. There is now accumulating evidence for a role of DSA-negative ABMR, but there is not much known about its pathophysiology or responsiveness to rejection treatment. The latter issue was addressed by Koslik et al. Evaluating 102 renal allograft recipients diagnosed with ABMR, among them 61 with detectable DSA, the authors studied the impact of multi-compound treatment, primarily based on apheresis and intravenous immunoglobulin (IVIG) on allograft outcomes. In case of persistent ABMR increase in maintenance immunosuppression or long-term application of IVIG was used. While late rejection diagnosis and positive C4d staining turned out to be independent risk factors for allograft failure, DSA status did not relate to graft survival. An interesting finding was that DSA-positive recipients showed significantly better allograft survival after long-term IVIG than patients with DSA-negative ABMR. Conversely, the latter exhibited better responses to intensified maintenance immunosuppression. Moreover, there are individual histological and molecular phenotypes of ABMR that may respond differently to treatment. In this context, Sazpinar et al. provide a detailed evaluation of 16 patients presenting with pure chronic active ABMR. The authors evaluated the expression of predefined rejection-related transcripts using NanoString™ technology and evaluated biopsy results in relation to clinical outcomes. In this preliminary small study, treatment responsiveness of ABMR was associated with the extent of microvascular inflammation and transcriptome changes in NK cell and endothelial cell associated genes. Cell therapies treating late ABMR are emerging including the transfer of mesenchymal stem cells (MSC). Still, these therapies might be accompanied by serious side-effects in individual patients as described by Večerić-Haler et al. They describe a single patient case with late ABMR treated with autologous MSC within a study and the patient developed life-threatening symptoms mimicking capillary leakage syndrome, which only resolved after explantation of the kidney graft. The authors speculate that Parvovirus B19 might have mediated the life-threatening condition. These data point to the fact that viral infections might be transferred *via* cell therapies which result in life-threatening complications for the patient.

There is definitely a need for well-designed randomized prospective trials to clarify the efficacy of new treatment approaches that are currently in the pipeline. Trial design in ABMR, however, is a challenge, partly because of the requirement of large patient numbers and long periods of follow-up to demonstrate meaningful differences in allograft survival. In a retrospective monocentric study, Borski et al. confirmed a strong impact of late ABMR on renal allograft survival, demonstrating a 93, 64, 53, and 15% unadjusted overall allograft survival at 1, 3, 5, and 10 years after index biopsy, respectively. In search of surrogate endpoints that allow for accurate prediction of graft survival, they found a strong predictive value of early eGFR decline. An eGFR loss of 1 ml/min/1.73 m^2^ per year was thereby associated with a 10% (12-month slope) to up to 30% (24-month slope) increase in the risk for future allograft loss. These data strongly support the utility of calculating eGFR slope as a surrogate endpoint of graft failure in ABMR trials. Moreover, in search of biopsy findings that predict transplant outcomes after ABMR diagnosis, Piñeiro et al. studied a retrospective cohort of 90 patients treated for active ABMR, exploring the clinical relevance of persistent inflammation detected in follow-up biopsies. Following treatment with plasma exchange, IVIG and rituximab, microvascular inflammation persisted in 71% and tubulitis in 19% of the biopsies. Persistent inflammation, even despite not strictly meeting any of the Banff rejection categories, was found to strongly associate with graft failure. Retreatment of patients with persistent inflammation was thereby associated with a better prognosis than in untreated patients. Finally, Novotny et al. studied 72 renal allograft recipients exhibiting vascular rejection (intimal arteritis), in association with TCMR or ABMR features (microvascular inflammation). The authors found that intimal arteritis in conjunction with ABMR was a significant risk factor of transplant glomerulopathy in follow-up biopsies, regardless of DSA status. Among DSA-positive patients with intimal arteritis and microvascular inflammation, resolution of rejection was less frequent (27% as compared to 58 or 90% in patients with phenotypes of intimal arteritis associated with DSA-negative ABMR or TCMR, respectively).

## Advances in cardiovascular disease and infections after kidney transplantation

Alloimmune responses are causative for kidney graft loss in a valuable number of our kidney transplant recipient. Still, cardiovascular events as well as infections are major causes of graft loss as well as death with a functioning graft after kidney transplantation (11). Thus, strategies to improve outcome after kidney transplantation in these areas are of critical importance to improve graft- and patient-survival. In the Research Topic Advances in kidney transplantation in Frontiers in Medicine published articles further add important new information in the field of cardiovascular disease and infections after kidney transplantation. Végh et al. showed nicely that blood pressure monitoring by 24-h measurements (ABPM) as well as vascular stiffness correlated with the GFR in the long term follow up of pediatric kidney transplantation recipients. Interestingly, there was no correlation of vascular stiffness and ABMR 2 years after transplantation, which points to the fact that CV health is of crucial importance for long-term function of grafts. Protection and/or of vascular calcification is an important step toward an improvement of cardiovascular survival in the chronic kidney disease population including kidney transplant recipients. Supplementation of magnesium has been proposed to be beneficial in treating vascular calcification in animal models (12). In line, low serum-magnesium levels have been associated with an increased cardiovascular risk and all-cause mortality in the general population (13). Contrarily, Lahav et al. show in their article in Frontiers in Medicine that serum-magnesium levels correlated inversely with all-cause and cardiovascular mortality in their cohort of kidney transplant recipients. Furthermore, magnesium supplementation did not show a benefit, but was rather associated with worse cardiovascular outcomes. Still, these outcomes might be biased by an improved graft function, which is associated with lower magnesium levels (14). Clearly interventional studies are needed to evaluate whether magnesium supplementation is beneficial in CKD patients as well as kidney transplant recipients. Since post-transplantation diabetes mellitus is one of the most important factors in increasing cardiovascular mortality in kidney transplantation recipients, studies addressing this issue are of outmost importance. Yin et al. published a study enrolling 105 kidney transplant recipients, who will be treated with placebo, metformin or empagliflozin. The two drugs will be tested for safety, effectivity, and tolerability in the kidney transplant population. The primary end point is the change in the visceral-to subcutaneous fat area evaluated by MRI as well as inflammatory parameters. This study critically addresses the problem of obesity and PTDM in the kidney transplantation cohort with the great opportunities of new therapeutic drugs such as SGLT-2 inhibitors, which have been shown to improve kidney function and cardiovascular mortality in the CKD cohort (15). Still, they also hold great potential in the kidney transplantation population, where only few studies exist evaluating these drugs (16, 17).

Infections are an important factor of graft and patient loss in the kidney transplantation population. Rare infections occur in our immune suppressed population such as shown in a recent case report of a patient developing malacoplakia due to the infection with a multi-resistant *E. coli* and *Cryptococcus albidus*. Both colonized in the transplanted kidney. Yan et al. further show that metagenome sequencing can be utilized as an additional diagnostic tool complementing pathogen detection especially in transplant recipients with unusual infections. In contrast, BK Polyoma virus infection is a frequent problem in kidney transplant recipients associated with an increased probability for graft loss. The study by Omić et al. showed that kidney transplant recipients with an insufficient decrease in BK Polyoma virus titers in the blood have a significantly increased risk for graft loss. Thus, not only patients with an initially high BK viral load, but also patients without a rapid decrease in BK viral load need to be closely followed. Omic also show that leflunomide did not improve GFR in their patient cohort but had the ability to fully clear the virus in a greater number of patients. Nevertheless, the only sufficient way to limit BK polyoma virus infection currently seems to be the reduction of immunosuppression. The COVID-19 pandemia put our kidney transplant cohort into severe risk for developing critical COVID-19 due to chronic immunosuppression and coexisting conditions (18). Dedinská et al. add to this evidence by providing retrospective, multicenter analysis of 186 kidney transplantation patients with COVID-19. Obese and patients >59 years are of high risk to develop critical COVID-19 illness. Corticosteroids more than 7.5 mg/day as well as HLA-DQ2 seem to be protective factors in their cohort of patients but need to be confirmed in larger trials.

Finally, a study on sex differences between start of kidney replacement therapy and inclusion on the waiting list for kidney transplantation has been published in this Research Topic of Frontiers in Medicine. Hödlmoser et al. show compelling sex disparities in both the US as well as in Austria with a significant higher chance for men to be listed for kidney transplantation. Even though the differences decrease over time, the gap persists especially for older women. Thus, we need to further understand the causes of sex disparities to set up ways to overcome them.

## Author contributions

KE, GB, MB, and OV: wrote, reviewed, and approved the manuscript. All authors contributed to the article and approved the submitted version.

## Conflict of interest

The authors declare that the research was conducted in the absence of any commercial or financial relationships that could be construed as a potential conflict of interest.

## Publisher's note

All claims expressed in this article are solely those of the authors and do not necessarily represent those of their affiliated organizations, or those of the publisher, the editors and the reviewers. Any product that may be evaluated in this article, or claim that may be made by its manufacturer, is not guaranteed or endorsed by the publisher.
